# A Path to Inclusiveness – Peer Support Groups as a Resource for Change

**DOI:** 10.1177/10598405221085183

**Published:** 2022-03-15

**Authors:** Ann Jeanette Heitmann, Lisbeth Valla, Elena Albertini Früh, Lisbeth G Kvarme

**Affiliations:** 60499 ^1^Oslo Metropolitan University, Pilestredet 32, N-0167, Oslo, Norway

**Keywords:** Bullying, Solution-Focused-Approach, Schoolchildren, Supportgroups, School nurse

## Abstract

Being bullied is associated with anxiety, depression symptoms, and long-term negative health outcomes. The aim of this qualitative pilotstudy was to explore bullied children's experiences of support groups and how participating in a group affected the children. The sample consisted of 24 children aged 11–13 years. Four of them were bullied, while 20 participated in support groups. Individual and focus group interviews were conducted. The main theme identified was that support groups provide an opportunity for change and can help children to be included among peers. The changes were achieved through encouragement and support from peers. The children participating in the support groups reported a feeling of being selected. The groups provided fellowship, and an opportunity for change. Both getting support from and being part of a support group contributed to inclusion, strength, and valuable experiences. The findings suggest that a systemic approach to bullying is advantageous.

## Introduction

Being bullied is associated with anxiety and depression symptoms (Ringdal et al., 2021), and victims of bullying may have long-term negative health-outcomes (Wolke & Lereya, 2015). Children who are frequently bullied are more likely to use mental health services during childhood (Finpå et al., 2017), adolescence and also in midlife (Evans-Lacko et al., 2016). Bullying interferes with functioning in the peer group (Kaufman et al., 2019), causes shame in the victim, in those who witness the bullying, and in those who actively bully other children (Jennifer & Cowie, 2012). A good psychosocial environment in school is paramount to reduce the burden associated with mental health disorders, especially in young people (Arango et al., 2018). Peer support contributes to a more positive school community and can also help bullied children to improve their situation (Cowie & Smith, 2010). Schools and mental health professionals need to demonstrate care for bullying victims (Kennedy, 2021). The Norwegian School Health Services’ aim is to collaborate with schools to create a good psychosocial environment for children, promote good mental and physical health, facilitate good social and environmental conditions, and prevent disease and injury (Norwegian Directorate of Health, 2019). In the Nordic countries, the prevalence of bullying has previously been reported to be about 14.7% (Krusell et al., 2019). Despite appropriate actions and practices having been implemented to prevent bullying and create a good environment in schools (Eriksen & Lyng, 2018a; NOU, 2015) bullying still affects around six percent of Norwegian children and young people (Wendelborg, 2020).

## Background

There are a variety of definitions of bullying. Partnership Against Bullying (2021), a coalition of 14 Norwegian national organizations, defines bullying as repeated negative behavior aimed at someone who cannot defend themselves. Bullying can be differentiated from other types of peer aggression by four key characteristics: frequency, intensity, power imbalance, and goal-directedness (Volk et al., 2015). Lund and Helgeland (2020) define bullying as an expression of social marginalization (Lund & Helgeland, 2020). This definition reflects a shift in focus from individual characteristics to social processes, where exclusionary mechanisms in children and young people's communities create a fertile ground for bullying. This perspective forms the basis for this article. Bullying is a complex and systemic issue (Waasdorp et al., 2019) that needs to be addressed at a broad system level (Fazel & Newby, 2021).The Bronfenbrenner bioecological model provides a framework for understanding the impact of a child's environment on the consequences of bullying. The microsystem refers to structures children are in direct contact with, such as family, peers, and the community. The interaction between the microsystems occurs in the mesosystem, such as the interaction between the school and family (Bronfenbrenner, 1979). The exosystem encompasses aspects of structures within the microsystem that may indirectly affect a child. The macrosystem includes social, economic, or cultural ideologies that affect the child's environment. This theory fundamentally describes how various systems influence a child's behavior in bullying situations (Banks et al., 2019). Ttofi and Farrington`s (2011) meta-analysis of 29 longitudinal studies recommended that new anti-bullying programs should take a systemic approach. Social inclusion is fundamental to learning important social skills, understanding the systemic interaction in the peer group, and developing skills relating to important aspects of children's lives (Veland et al., 2015).

### A Solution-Focused Approach

This study used the Solution-Focused Approach (SFA) to intervene with children who were bullied. (Kvarme et al., 2016; Young, 2009). SFA is a social constructionist approach established to facilitate change by highlighting students’ goals, strengths, and resources, and to identify exceptions to the problem. The SFA emphasizes people's personal strengths and successes as valuable learning experiences, acknowledges that people can change and presumes that shifting from being a victim to taking a stand creates optimism, self-belief, and trust that a situation can be altered (Young, 2009). It is an effective treatment strategy for a wide variety of behavioral and psychological outcomes (Gingerich & Peterson, 2012), and forms the basis for the support group intervention. The most important elements of SFA are identifying exceptions, scaling questions, promoting future-directed thought orientation, prompting children to find exceptions that highlight small successes, and using the miracle question to help children visualize their future as if the problem no longer existed (Schmit et al., 2016).

## Aim

The aim of this pilot study was to explore bullied children's experiences of support groups and how participating in a group affected the children.

## Methods

### Study Design

This is a qualitative study with an exploratory design and a phenomenological hermeneutic mode of understanding. Data were collected through interviews with bullied children and support group members.

#### Participants and Data Collection

The study sample consisted of 24 children, aged 11–13 years, from one urban and one suburban school. They were recruited by teachers and school nurses. Four of them, three girls and one boy, experienced bullying, and 20 participated in support groups. The school nurse or teacher distributed information letters about the study and consent forms to the children and their parents. After obtaining written informed consent from both children and their parents, the study commenced. Data were collected at the children's school during school time in January and April 2021. Individual interviews with children who experienced bullying and focus group interviews with support groups were conducted by the first author. A recorder was used to collect the data. The individual interviews lasted for approximately 40 min, focus group interviews for approximately 45–60 min.

### The Support Group Intervention

Support groups are based on SFA and have largely succeeded in helping children regain mastery and social inclusion (Cowie, 2011). All the schools participating in this study received information and four days training before start-up. Teachers and school nurses were invited, 15 teachers and school nurses volunteered to join the training. From the two schools included in this particular study three teachers and one school nurse participated.

The training consisted of 2 days with SFA and 2 days training on how to run the groups. In addition, the trainers received a manual for implementation. Written information to all parents with information about support groups was presented and parents were given the opportunity to withdraw their child from participation. Prior to support group intervention, the child had typically contacted a school nurse or teacher to help combat bullying or exclusion. Having established a relationship, the teacher or school nurse assessed whether they thought support group intervention was well-suited for the child. Together, the teacher or nurse and the child agreed on between five and seven peers who they considered suitable to join the group. Who is suitable may vary regarding to the child's challenges. In this study, the support groups consisted of both girls and boys. The children's parents received written information and signed to consent to participation before start-up. When the selected children were asked to join in the support group, they were briefly informed about the situation of the bullied or excluded child, asked if they had experienced similar situation, and if they wanted to help. The school nurse or teacher held weekly consultations with the support group, with each session lasting for approximately 30 min. Members of the support group were encouraged to suggest helpful ways to help the bullied or excluded child. They looked for exceptions and progress, and the school nurse gave the children compliments and encouragement, in line with SFA. In this way, the group members were actively included.

The bullied child did not join the group, he or she had individual consultations with the school nurse or teacher. All consultations took place during school-hours and were held in accordance with the SFA.

### Analysis

Graneheim and Lundman`s (2004) model for qualitative content analysis was used to analyze the data (Graneheim & Lundman, 2004). The interviews were transcribed verbatim by the first author, listened to and read through several times to gain an understanding of the material as a whole. The first author identified meaning units and condensed them as sub-themes and themes. The themes were examined to identify similarities, then sorted and abstracted into main themes. At the end of the analysis process, all authors discussed the results until consensus was achieved. The analysis process was in line with the hermeneutic circle and Watzlawick et al. (1967) understanding of human communication (Watzlawick et al., 1967). The analysis process was thereby characterized by the work on the themes and main themes, while the interviews were listened to for he latent content in our understanding of the children's descriptions. Pre-understanding among the authors also has the potential to influence the process of analyzing the data. All the authors are experienced school nurses with expertise in talking to children. The first author is also a family therapist, with personal experience of running support groups. (Table 2)

**Table 2.** Examples from the Analysis Matrix.

Quotes	What is being said	Sub-theme	Theme
A bullied child: “Usually, I have been very lonely at break times, walked around alone and hid myself. But after the group started, they have asked me before break time if we can play together, so it is a very good arrangement. My school day is simply much better.”	She was lonely before and used to hide. Now the group members invite her to play at break times. She likes the arrangement. Her school days are much better.	Loneliness To be included Outcomes of the support group intervention	**From lonely to included**
A bullied child: “At first, I thought it was a bit difficult to tell people about my loneliness, since I am not used to telling anyone when I feel bad, so I felt insecure. Now I know that asking for help is a good thing. I feel so much better.”	Difficult to tell people about loneliness, not used to opening up. Felt insecure It helped Feels better	To open up about difficulties The situation changed and she feels better.	**The value of opening up about difficulties**
A bullied child: “Before, I used to be angry at my peers, and I didn’t like to play with the others. But after the group started inviting me, things changed. Also, I was given a specific task by the social teacher. I was to look for nice things that happened. Now I feel that I have changed. I am not as angry at my peers as I was before. It feels good. The biggest change is within myself actually.”	Used to be angry at his peers The group invited him to play Task: look for nice things that happened. He changed. Biggest change is within himself.	Invitations and a new task made him change within himself.	**An opportunity for change**
A support group member: “Participating in something that makes a peer happy makes me feel good. For me, being selected to join a group like this is confirmation that I am a kind person, that people trust me and want me to contribute.”	It feels good to participate and make a peer happy. Being selected is confirmation that she is a kind person.	Feels good to be selected. Confirmation that she is a good person.	**A feeling of being selected**
A support group member: “She has always been very sensitive, and I have felt so much responsibility for her. She has often expressed a feeling of being lonely, but I have seen that she has always been playing with someone, and everyone has talked to her and we have had a lot of fun together. Suddenly she just goes somewhere and cries and says that she feels alone and that we have kept her at a distance. I think it has been very tiring.”	Felt responsible for a peer. She expressed a feeling of being lonely, but she has always been playing with someone. She says that we keep her at a distance. It has been very tiring.	Feels responsible, but she sees the situation differently. She feels tired.	**A demanding role to play**
A support group member: “I didn’t dare to go to him, I was afraid of what the others might say. But after the support group, I thought: Well, I am in this group now, I feel stronger because of them. So now I have asked him to join us. It felt so good to have more courage.”	Before, he was afraid of how peers might react. The group makes him feel stronger. A good feeling to have more courage.	He was afraid before, now he feels stronger. It feels good to have more courage.	**Gaining strength and courage**

### Interview Guide

An interview guide was prepared before the data collection started (Table 1). In line with an exploratory design, the questions were open-ended to gain insight into the participants’ own experiences, without guidance. The questions contained elements of the SFA and the children were encouraged to add comments of their own. An informal and relaxed atmosphere was created to make the children feel comfortable. The school nurse was available for the children if they needed support after the interviews.

**Table 1.** Interview Guide.

Main questions for the individual interviews:
How did you experience having a support group? On a scale from 1–10 (where 10 is best), how did you experience your school days before the support group started up? (Please feel free to elaborate your answer) On a scale from 1–10, how do you experience your school days now? (Please feel free to elaborate your answer) Can you describe in what way the support group has made a difference to you? Can you share some examples of what the support group has done? Is there anything else you would like to share from this experience? Is there anything that could have been done differently, that you miss?
Main questions for the focus group interviews:
How did you experience being part of a support group? Can you describe how the support group has made a difference? On a scale from 1–10 (where 10 is the best), what was the classroom environment like before the support group started up? (Please feel free to elaborate your answer) On a scale from 1-10, what is the classroom environment like now? (Please feel free to elaborate your answer) How has supporting a peer affected you? Is there a specific experience related to being part of the group that you would like to share? Is there anything that could have been done differently, that you miss?

## Ethical Approval and Considerations

This pilot study was approved by the Norwegian Social Science Data Services (NSD) and the Regional Ethical Committee*.* Research on children requires a strong awareness of their vulnerability. The relationship between children's valuable knowledge and their vulnerability had an essential place in the ethical assessments that were carried out. The participants and their parents received information about the aim of this study and the data collection procedures before signing up for participation. They were also informed that their participation was voluntary, would not affect the help they received, and that they could withdraw from the study at any time without consequences. Written informed consent to participate was obtained from all participants and their parents before the interviews were conducted. The data were anonymized and stored safely on an encrypted memory stick that was stored securely, to ensure the children's anonymity. Children in the support groups were asked to contribute to a respectful atmosphere and keep the information that was collected to themselves. After the interviews were conducted the findings were summarized for the children and they were encouraged to correct if something had been perceived incorrectly. A safe atmosphere was facilitated for the children, as this increases the trustworthiness of the data (Malterud, 2012). All the children were encouraged to participate in the conversation. However, in a group discussion the participants influence each other, and some may be afraid to express their thoughts. As a contribution to make sure everyone was involved, questions were asked to each participant. All data will be deleted upon completion of the project.

## Results

The following main themes were identified when analyzing the data collected from the individual interviews: from lonely to included; the value of opening up about difficulties; an opportunity for change. From the focus group interviews, the following themes were identified: a feeling of being selected; a demanding role to play; gaining strength and courage. The results of the individual interviews are presented first, followed by the results of the focus group interviews.

### Individual Interviews with Bullied Children

#### From Lonely to Included

All the children described a feeling of loneliness. For different reasons they had no friends to play with. One of the girls said: *“I walked around alone and hid myself. I didn’t want to go to school, and I had no friends.”* After the support group began, her situation changed: *“My peers came and asked me before the break if I wanted to play with them. My schooldays improved.”* Another girl said that her best friend moved away, and after that she felt lonely. She didn’t know what to do about the situation, so she told the school nurse, who suggested a support group for her. The school nurse made her believe that would improve her situation, and it did. The support group members started to ask her how she felt, and they invited her to do schoolwork with them: *“It felt really good! Now I look forward to going to school, it`s a great improvement!”* she said.

The boy stated that all the fighting on the football field affected him and made him feel scared. He talked to the teacher about it and the support group contributed to great change: *“If there is a fight now, my peers help me. It makes me feel safer. I think all my peers like this change.”*

The children described the importance of having a friend and how the feeling of being included had made them feel better about themselves. Small changes, like someone greeting them in the morning and being invited to play at break times, had a great impact on their everyday lives. One of the girls found a new friend: *“One of the girls in the group and I, we play together every day, and we talk. We can talk about everything, and we share our secrets. She is the best friend I’ve ever had.”*

#### The Value of Opening up About Difficulties

The children reported feeling insecure when opening up about their difficulties, but they all experienced that doing so improved their situation. One girl said that she thought a lot about it before she spoke to the teacher: *“I’m not used to telling anyone when I feel bad, so I felt insecure. Now I know that asking for help is a good thing. I feel so much better,”* she said. One of the other girls had a similar experience. She used to keep her feelings inside her: *“When I try to say something, it just stops inside me, although I want to talk about it. Because of that I often keep it to myself and I try to find my own solutions.”* She felt insecure at first, but when she eventually opened up, her situation improved:

“I was a little scared. How much would the school nurse tell my peers? I was glad when she informed me what she was going to tell them. It made me feel more in control and safer, I was relieved.”

The children said that it took a lot of courage to open up. They were afraid that the support group would tell their peers about it, but fortunately they all experienced that they did not:

“They kept it a secret. Even the girl who can be rude, she didn`t say anything. I was so happy about that! I will recommend a support group to others who feel alone. Take a chance!”

#### An Opportunity for Change

**The bullied children had weekly consultations with their teacher or school nurse. These** sessions were based on SFA. The children said that they learned a lot about themselves. One girl was given a specific task by the social teacher: *“She gave me a challenge to do things that I haven`t done before, like playing with several peers at the same time.”* She always preferred being with just one friend at a time. When all her peers played together, she used to hide:

“When we were three girls playing together, I often felt excluded. Now, I have learned that it can be nice to play with more than one friend at a time. I have challenged myself to do something new and I like it! Sometimes I ask others to join in too, I never did that before. It makes me feel good about myself. To be honest, I am quite proud of myself.”

One of the boys was also given a task by the social teacher, he was to look for good things that happened during break times. This made him see things differently, he experienced a change within him: *“I feel that I have changed. I am not as angry at my peers as I was before. It feels good. The biggest change is within me actually.”*

This opportunity for change was based on a feeling of being listened to and taken seriously:

“I got to decide who I wanted to join in the support group. This gave me a feeling of control. I chose some that I knew quite well, and some that I wanted to get to know and felt it would be good to have in the group.”

Working with support groups offers an opportunity for change beyond the school situation. One of the girls shared that difficulties at home affected her and the way she met her peers: *“I often felt sad. We had some problems in my family which affected me greatly. After my situation at school improved, I fought a lot less with my sisters.”* When things improved at home, she came to school in the morning feeling better, which, in turn, affected her school days.

The children discovered that the support group also affected the social environment among their peers: *“The classroom environment has improved. We are not fighting as much as we did,”* one boy said. The extra attention from the teacher or school nurse also meant a lot. When the support group started up, the teacher and school nurse showed a lot of care:

“They paid attention to what we did during break times. If something happened, or I just wanted to tell them something, I knew I could talk to them and they would help me. It made me feel safer.”

The children explained that the changes had affected them greatly. One of the girls explained that she looked forward to going to school now: *“In the morning, I hurry to meet my new friend and go to school together with her.”* She wanted to recommend a support group to everyone who feels excluded or wants a change: *“It might help you to feel better at school.”*

### Focus Group Interviews

#### A Feeling of Being Selected

Support group members expressed a feeling of being selected:

“Participating in something that makes a peer happy makes me feel good. For me, being selected to join a group like this is confirmation that I am a kind person, people trust me and want me to contribute.”

This feeling of being selected and able to make a difference was very important: *“It makes me so happy to see that I can make a change, I can contribute to improving how a friend feels at school. I can make her feel better!”* The girls had made a great effort to improve the peer`s situation: *“I have invited her to play with us, both at school and after. Sometimes she has slept over at my house. We had a great time together!”* The girls also said that they appreciated being included in the support group and explained how it made them feel better about themselves: *“I think we were chosen because she thinks that we are kind and can help.”*

The boys in the group said that they felt they were on an important mission:

“We were supposed to look after him during break times. And if anyone hurt him, we were going to support and help him. I liked it a lot. It felt good and it was quite fun.”

They confirmed what the bullied boy had shared about his situation, how his peers used to do mean things to him, especially on the football field. The boys were supposed to stand up for him, and they did. They also described how the new role had changed them: *“It feels good to say nice things to people, I do it often now actually.”*

#### A Demanding Role to Play

One of the girls in the support group explained that she was surprised when she was asked to contribute to the support group. She felt a great responsibility for her peer, but she was tired of being the one who was supposed to help all the time. She was also in doubt about her peer's explanations:

“She has always been very sensitive, and I have felt so much responsibility for her. She has often expressed a feeling of being lonely, but I have seen that she was always playing with someone, and everyone has talked to her and we have had a lot of fun together. Suddenly she just goes somewhere and cries and says that she feels alone and that we have kept her at a distance. I think that has been very tiring.”

She said that it was difficult to understand how her peer felt. In her opinion, no one had bullied her. She felt naughty telling us about this, she said. She wanted to be a good person, but it was also important for her to say things straight out: “*Like Ìm not a good person since I say things straight out. It makes me feel bad,”* she said*.* The school nurse understood that the responsibility weighed heavily on her and that she needed a break. She kept some distance for a while, which helped them find their way back to friendship.

Some of the girls in another group described how much they liked to help, but also felt that it cost them a lot. They chose to prioritize their friend and opted out of things they themselves wanted to do. They found it hard in the beginning, because they liked to play with several friends at the same time. The bullied girl found it hard because of bad experiences. Luckily the situation changed: *“Now it`s much better. She can play with several people at a time now.”*

The girls suggested that the group leader should pay more frequent attention to how the group members felt: *“It would be good if they asked a little about how we felt when we try to help our peer.”*

#### Gaining Strength and Courage

The boys said that being part of the support group had given them strength and courage:

“Before, I never dared to talk to the girls. Since the fighting stopped on the football field, they have joined us. Now I even play with them. Also, if anyone says something bad, I ask them to stop. I never did that before.”

One of the other boys described how much he enjoyed participating in the support group:

“It has been so much fun. I’ve got a new friend! He is so nice. It`s so much better to be kind than mean, because when you are mean you hurt people.”

He explained how they felt a strong sense of fellowship and felt safer because of the group:

“Before, I was shy, I didn`t ask anyone to join a game. Now I feel that I’m part of a club, like we are all in this together, now I have more courage to invite others. It feels good.”

One of the girls said that many of her peers, including herself, had been afraid of being bullied themselves, so they didn`t dare to invite her to play: *“I think my peers are glad that we made a change. I think many of them are relieved.”* The girl who felt it was hard to be the one who was always expected to help had several bad experiences of interventions. She felt that, no matter how much she tried, it did not help. Some of what the school had tried before had only helped to conceal the problem. With the support group she had a different experience: *“The support group made a difference! Finally, something works! Now it feels good to be the one helping.”*

## Discussion

The main theme identified in this pilot study is that support groups offer an opportunity for change and can help children to be included among their peers. The children experienced the value of opening up about difficulties. Both getting support and being part of a support group contributed to inclusion, courage, and strength. Children who contributed to the support groups felt selected, but also experienced that it could be a demanding role to play. The significance of taking a systemic approach to bullying and the role of school nurses or teachers as important contributors to change was very clear. Children in this study described how contributing to a support group made them feel selected. They felt a strong fellowship and were willing to sacrifice their own wishes to help their peer, but the study also showed that it can be challenging to contribute. One of the girls explained how it was difficult for her to be part of the support group, she was tired of being the one who was supposed to help, and she felt naughty telling us about this. These findings are in line with previous research in which support group members explained how they felt unsure of their role and experienced conflicts (Kvarme et al., 2016). A recent study, (Waasdorp et al., 2019) on the significance of individual and classroom factors in relation to having sympathy for victimized peers, highlighted the importance of knowledge about factors that influence peer sympathy for bullied children, e.g., girls and boys need different support to increase sympathy. According to Waasdorp et al. (2019) sympathy is linked with defending behavior (Waasdorp et al., 2019). However, it is important to distinguish the difference between sympathy, empathy and compassion, and how it affects the interpretation of both the literature and the children's statements. While sympathy is described as a pity-based response, empathy acknowledges an individual's suffering through emotional resonance, and compassion seeks to address the suffering and needs through relational understanding and action (Sinclair et al., 2017). When a child feels tired of being the one to help, together with a feeling of being naughty when telling about it, it may indicate a feeling of shame. Brown (2006) describes shame as “an intensely painful feeling or experience of believing we are flawed and therefore unworthy of acceptance and belonging.” The participants in her study described shame as a feeling of being trapped and powerless, and the feeling of empathy as the opposite, a feeling of connection, power and freedom (Brown, 2006). Knowledge and capacity to integrate these considerations in the support group approach is important.

It has been speculated that defending victims is stressful and can contribute to poor mental health. Recent studies have not supported those findings, however (Malamut et al., 2021; Sjøgren et al., 2020). A longitudinal analysis among 4086 youths showed that, for youths with social resources, defending victims can have a protective effect on mental well-being. These findings are important for further development of support groups as a tool to combat bullying, and they emphasize the importance of awareness of who should participate in the groups, duration of the follow-up and the importance of knowledge and capacity to follow up the participants in a professional manner.

The SFA is essential in work with support groups, since it provides an opportunity to look for solutions rather than focusing on difficulties. Looking for the children's strengths instead of their weaknesses provides an opportunity for a new understanding of oneself (Young, 2009). Both bullied children and support group members described how they changed through participating, or through support, challenges, and encouragement from the school nurse. To overcome a bystander role and become a person who stands up for others can influence a child's self-esteem to a great extent (Salmivalli, 2010). The SFA provides an opportunity to focus on the children's personal strengths and successes as valuable learning experiences (Young & Holdorf, 2003). In this pilot study, the participants stated that they had gained strength and courage through inclusion and fellowship. The support group had given them an opportunity to change. According to Young (2009), the support group approach, with the SFA at its core, can be regarded as a brief therapy. However, this presupposes that the school nurse or teacher has the competence in how to use it in conversations with the children.

### A Systemic Approach to Bullying

The importance of peer support and the child's social context was apparent in this pilot study. Bronfenbrenner's model helps us to understand how the interaction between the different system levels affects the child (Bronfenbrenner, 1979). It may also contribute to understanding why bullying is a complex group phenomenon (Salmivalli, 2010) that needs to be addressed at a broad system level, including peers, family, and teachers, and takes the overall challenges of inclusion and acceptance into consideration (Fazel & Newby, 2021). Having a friend and support from peers can protect children against bullying (Kendrick et al., 2012). Social support from peers contributes to lower levels of anxiety and depression symptoms and higher scores for mental well-being (Ringdal et al., 2021).

In this pilot study, the children's situation at home proved to be an important factor. The significance of this was supported by a study from 2020 where collaboration with parents was found to be essential to stop bullying. The fact that parents teach their children to stand up for others had a strong connection with their knowledge and cooperation with the school (Banks et al., 2019). However, there is some uncertainty about the importance of the children's home situation. Veland et al. (2015) found a weak correlation between children's socioeconomic situation, parenting-style, and the children's perceived inclusion in school (Veland et al., 2015). Kennedy (2021) refers to research that claims that victims of bullying are more likely to be treated harshly by their parents, have a chaotic home life and to have experienced trauma in their lives (Kennedy, 2021). These findings emphasize the importance of interventions to provide care for children who experience exclusion, loneliness, and bullying.

Anti-bullying programs should take a systemic approach and include both the social environment at school and the children`s families (Ttofi & Farrington, 2011). According to Menesinia and Salmivalli (2017), involving parents seems to strengthen the effects of programs (Menesini & Salmivalli, 2017). School nurses have a key role in safeguarding children's mental health and they are in an ideal position to initiate interventions to prevent school bullying (Jacobson et al., 2011). Interdisciplinary programs are more likely to succeed (Ttofi & Farrington, 2011) and school nurses are in a unique position to lead the interdisciplinary collaboration against bullying (Cooper et al., 2012). Results from our pilot study indicated that support groups may help children to be included among peers and provides an opportunity for change for both bullied children and children participating in a support group. Thus, it may indicate that this is a method that can be useful for school nurses and teachers working with vulnerable children. A classroom environment with high levels of teacher–student closeness facilitates greater sympathy for bullied peers (Waasdorp et al., 2019). A quantitative study from England, including 1648 children aged from 8 to 12 years, published in March 2021, highlighted the importance of regarding inclusion as the responsibility of the whole school, family, and community rather than as an individual challenge (Fazel & Newby, 2021). However, there is a need for more studies to investigate the effects of support groups as a tool against bullying and exclusion.

## Limitations

The present pilot study provides insight into how support groups can help bullied children, but the results must be interpreted in light of methodological limitations. The data were collected through individual and focus group interviews. Individual interviews provide thorough insight into the children's experiences, but certain aspects need to be considered. Children are vulnerable in encounters with adults and bullying is a sensitive subject with the potential to evoke strong emotions. The interview guide was prepared in line with an exploratory design, in order to gain insight into the participants’ own experiences. However, more specific questions might have provided more in-depth data. The researcher's experiences, competence, and discourses may affect both what emerges in the interviews and how it is interpreted afterwards. This must be taken into account when interpreting and analyzing the children's statements, and also when assessing the results. Results from qualitative studies reflect the participants’ voices and the researcher's reflexivity (Creswell & Poth, 2018). Focus group interviews differ from individual interviews in their context, and provide different knowledge (Malterud, 2012). The children are given an opportunity to inspire, but also influence each other's experiences and explanations. The fact that the children were only interviewed once means that there was no opportunity to follow up the results over time. However, the results correspond with previous research and may be transferable to similar contexts involving schoolchildren of the same age.

## Recommendations for Future Research

Further research on support groups is important for the development and further implementation of this intervention in schools. There is a need for effect studies, such as RCT and longitudinal studies, that can follow up the results, to see whether they persist. Programs and practices need to be scaled up and sustained over time to have the desired long-term outcomes (Fixsen et al., 2017).

## Conclusion

The main finding in this pilot study was that support groups may provide an opportunity for change. Bullied children described feeling vulnerable when they opened up about their difficulties, but it helped them to make a change and be included among their peers. Children who contributed to support groups described feeling selected, but also experienced that it could be challenging to help a peer. Both bullied children and children in the support groups reported that they gained strength and courage through participation. The findings suggest taking a systemic approach to bullying, and they emphasize peers as a resource for change, together with close follow-up from school nurses and teachers.
